# Effects of Pretreatment Technologies on the Physicochemical Properties, Fatty Acid Composition, Active Components, Antioxidant Capacity, and Volatile Flavor Compounds of Cold‐Pressed *Litsea cubeba* Kernel Oil

**DOI:** 10.1002/fsn3.72138

**Published:** 2026-07-23

**Authors:** Xinsheng Xiao, Yiyang Cao, Jialing Li, Zhenfeng Jiang, Jinhua Shao, Hongyu Jiang, Liyan Jiang

**Affiliations:** ^1^ College of Chemistry and Bioengineering Hunan University of Science and Engineering Yongzhou Hunan China; ^2^ Hunan Yaozhen Grain and Oil Co. Ltd, Jianghua Yao Autonomous County Yongzhou Hunan China

**Keywords:** cold pressing, *Litsea cubeba* kernel oil, pretreatment technology, volatile flavor compounds

## Abstract

This study compared the differential effects of various pretreatment technologies (stir‐frying, roasting, infrared, and microwave) on the quality and flavor characteristics of cold‐pressed *Litsea cubeba* kernel oil (CLCKO). Results indicated that different pretreatment methods had no significant impact on the fatty acid composition of the oil but significantly influenced its physicochemical properties, bioactive components, antioxidant capacity, and flavor profile. Among these, the infrared‐pretreated group exhibited the highest oil yield (17.19% ± 0.03%), higher total phenolic content (199.33 ± 6.92 mg/100 g), and demonstrated an excellent scavenging rate of 2,2‐diphenyl‐1‐picrylhydrazyl (DPPH) radicals. Concurrently, the key aroma compounds in the infrared‐pretreated oil were dominated by (−)‐β‐caryophyllene (sweet, woody, and spicy), collaborating with key aroma components such as citronellal, geraniol, and myrcene, significantly enhancing floral‐fruity, citrus, and peppery notes. These findings demonstrate that infrared pretreatment effectively enhances extraction efficiency, improves nutritional profile, and elevates flavor characteristics in CLCKO, providing valuable insights for developing high‐quality *L. cubeba* by‐products.

## Graphical abstract

This study demonstrates that pretreatment techniques exert a substantial influence on the quality and flavor profile of cold‐pressed *Litsea cubeba* kernel oil—enhancing oil yield, increasing the concentration of bioactive compounds, and improving flavor attributes. Among the various pretreatment methods evaluated, infrared pretreatment emerges as a highly efficient approach with considerable potential for industrial application.

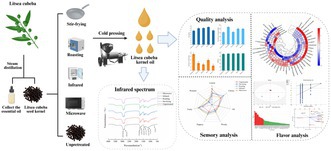

## 1 Introduction

*Litsea cubeba* , commonly known as mountain pepper, wood ginger, or mountain pepper tree, is a plant belonging to the *Litsea* genus within the Lauraceae family. It is widely distributed across most regions of China and countries in Southeast Asia (Zhou et al. 2020). Its fruits are primarily used for essential oil extraction, but the average oil yield is only 4%. After essential oil extraction, a large amount of kernel residue is produced (Qiu et al. 2021). Notably, the discarded kernel residue holds significant potential for utilization. *L. cubeba* kernels (LCK) are rich in oil content, ranging from 25% to 40% (Xiao et al. 2023; Zou et al. 2023), and predominantly composed of medium‐chain fatty acids (Zhuang et al. 2018). This profile holds significant application potential in surfactants, biodiesel, and bio‐lubricants (Qiu et al. 2021). Therefore, deep processing of *L. cubeba* kernel oil (LCKO) is crucial for enhancing its value and expanding its applications in the cosmetics and fine chemicals industries (Li, Wang, et al. 2024; Li, Hu, et al. 2025).

Currently, LCKO extraction primarily relies on mechanical pressing (Yuan et al. 2006) and solvent extraction (Zhuang et al. 2018; Li et al. 2016). While widely used, these methods have notable limitations: mechanical pressing is divided into hot and cold pressing, with cold pressing being the safest method for oil extraction from oilseeds. However, despite its high safety, it yields low oil extraction rates (Masoodi et al. 2022). Solvent extraction offers higher yields but suffers from high energy consumption and prolonged processing times (Hao et al. 2022). Research indicates that pretreating oilseeds like perilla seeds (Huang et al. 2023), canola seed (Li, Zhu, et al. 2025), and sunflower seeds (Wu et al. 2025) effectively disrupts cell wall and membrane barriers while loosening cellular structures, thereby enhancing oil extraction rates. Furthermore, pretreatment can improve oil quality and impart distinctive flavors. For instance, stir‐frying at the optimal temperature and duration yields Camellia seed oil with the most intense, fresh, and authentic tea oil–characteristic aroma (Yang, Wei, et al. 2024). Roasting helps produce roasted aromas but may affect oil antioxidant capacity (Lu et al. 2024). Infrared pretreatment improves perilla seed oil quality (Huang et al. 2023) and promotes the formation of specific volatile flavor compounds in rapeseed oil (Yu et al. 2021). Microwave treatment facilitates cell rupture and active compound release while reducing harmful oxidative byproducts (Shyam et al. 2024). Therefore, to overcome low oil yield and improve oil flavor, appropriate pretreatment measures can be applied before cold‐pressing *L. cubeba* kernel oil (CLCKO).

However, current research on LCKO primarily focuses on optimizing extraction processes (Yang, Yang, et al. 2024), analyzing compositional components (Zou et al. 2023), and product development (Xiao et al. 2023). Studies investigating the differential effects of various pretreatment methods on the comprehensive quality attributes of CLCKO, including physicochemical properties, bioactive constituents, antioxidant capacity, and volatile flavor characteristics, remain largely unexplored. Therefore, this study systematically investigated the effects of four pretreatment technologies—stir‐frying, roasting, infrared, and microwave on the oil yield, physicochemical properties, fatty acid composition, and active components. It also compared and analyzed the flavor change patterns of CLCKO under different pretreatment methods. This study aimed to provide new insights for the high‐value utilization of LCKO and offer a reference for the development of other woody oil crops.

## 2 Materials and Methods

### 2.1 Materials and Reagents

*L. cubeba* fruits were collected from Huitong County, Huaihua City, Hunan Province (26°53′ N, 109°43′ E). Fruits were selected based on fullness, integrity, uniform size, absence of rot, and free from insect damage. The kernels obtained after steam distillation for essential oil extraction constitute the LCK material (moisture content: 8.74% ± 0.16%; oil content: 19.95% ± 0.29%).

The reagents used in this study are listed as follows. 37 fatty acid methyl ester standards, Sigma‐Aldrich Trading Co. Ltd. (Shanghai, China); petroleum ether, Anhui Tiandi High‐Purity Reagent Co. Ltd. (Anhui, China); n‐hexane (chromatography grade), VBS Corporation (USA); methanol, Fisher Scientific (USA); anhydrous ethanol, dichloromethane, Tianjin Zhiyuan Chemical Reagent Co. Ltd. (Tianjin, China); Potassium hydroxide, sodium hydroxide, anhydrous sodium sulfate, Tianjin Fuchen Chemical Reagent Factory (Tianjin, China); Ethyl ether, chloroform, glacial acetic acid, isopropyl alcohol, Hunan Huihong Reagent Co. Ltd. (Hunan, China); Isooctane, Tianjin Kemiou Chemical Reagent Co. Ltd. (Tianjin, China); Gallic acid, Phloretin, Tianjin Guangfu Fine Chemical Research Institute (Tianjin, China); Phenolphthalein, Tianjin Damiao Chemical Reagent Factory (Tianjin, China); p‐Anisidine, Shanghai Titan Technology Co. Ltd. (Shanghai, China); DPPH (1,1‐Dimethyl‐2,2‐diphenylhydrazine), Sigma‐Aldrich (USA).

### 2.2 Methods

#### 2.2.1 Preparation of *Litsea cubeba* Kernels

One hundred grams of *L. cubeba* fruit were placed into a 1 L flask, along with 600 mL of distilled water and 10 g of NaCl. Once the essential oil distillation apparatus had been securely connected, the mixture was brought to a boil. After the initial heating phase, the power of the electric furnace was adjusted to 800 W.

The distillation was maintained at a steady 80°C for a duration of 120 min. While the mixture was boiling, the volatile compounds transitioned into the vapor phase before condensing. Once the distillation had finished, the resulting extract was transferred to a separatory funnel.

The mixture was left to sit until the oil and water phases had completely separated. After the aqueous phase had been discarded, an appropriate amount of anhydrous sodium sulfate was added to the oil. The oil was left to dehydrate for 12 h. Finally, the mixture was filtered to obtain the pure, anhydrous *L. cubeba* essential oil (LCEO). The residue that remained in the flask after the extraction process was LCK.

#### 2.2.2 Sample Thermal Pretreatment

To prepare the samples for analysis, four distinct thermal treatments were applied to the LCK. First, 200 g of LCK were accurately weighed and then roasted in a preheated oven at 130°C for 20 min. In a separate trial, after another 200 g sample had been weighed, it was stir‐fried at a higher temperature of 180°C for a shorter duration of 15 min.

The third group of kernels underwent infrared pretreatment. Once 200 g of LCK had been measured out, it was subjected to infrared radiation at 140°C for a period of 40 min. Finally, for the microwave pretreatment, 200 g of LCK were weighed and then processed in a microwave reactor at a power setting of 640 W for 10 min. Unpretreated LCK served as the control group.

Samples (pretreatment groups and control group) underwent cold‐press extraction under the following parameters: temperature 50°C ± 5°C, pressure 50 MPa. After pressing, samples were centrifuged at 6000 rpm for 10 min. The supernatant oil was collected, sealed, and stored at −4°C.

$$\text{oil yield}\left(\%\right)=\frac{m}{M}\times 100$$

where *m* = kernel mass, g; *M* = *L. cubeba* kernel oil mass, g.

#### 2.2.3 Fourier Transform Infrared Spectroscopy (FTIR) Analysis

Infrared spectra were acquired using a Fourier Transform Infrared Spectrometer (Model IS10, Nicolet, USA). Within the spectral range of 500–4000 cm^−1^, infrared spectra were recorded at 4 cm^−1^ resolution with 32 scans per sample (Suri et al. 2019).

#### 2.2.4 Determination of Physicochemical Indicators

The method for determining acid value was established following the procedure outlined in GB 5009.229‐2016 “National Food Safety Standard (National Food Safety Standard 2025): Determination of Acid Value in Food.” The method for determining peroxide value followed the procedure to GB 5009.227‐2023 “National Food Safety Standard: Determination of Peroxide Value in Food” (National Food Safety Standard 2023). The method for determining anise amine value was assessed by referring to GB/T 24304‐2009 “Animal and Vegetable Fats and Oils: Determination of Anise Amine Value” (National Technical Committee on Grain and Oil Standardization 2009).

#### 2.2.5 Determination of Fatty Acid Composition

The fatty acid composition of CLCKO was determined according to GB 5009.168‐2016 “National Food Safety Standard: Determination of Fatty Acids in Foods” (National Food Safety Standard 2016).

#### 2.2.6 Determination of Active Components in CLCKO

##### 2.2.6.1 Total Phenolics

Following the method presented by Khattab et al. (2010), a standard curve was prepared and used to determine the total phenolics content of the sample.

##### 2.2.6.2 Squalene

Squalene extraction was performed according to GB 5535.2‐2008 “Determination of Unsaponifiable Matter in Animal and Vegetable Fats and Oils” Part 2 (National Technical Committee on Grain and Oil Standardization 2008): Hexane Extraction Method. Ten milligrams of unsaponifiable matter were dissolved in dichloromethane to a final volume of 10 mL. A 1.5 mL aliquot was filtered through a 0.45 μm organic microporous membrane for subsequent GC–MS analysis.

GC Conditions: INERTCAP quartz capillary column (30 m × 0.25 mm × 0.25 μm), splitless injection, injection volume: 1 μL; Temperature program: Column oven temperature held at 80°C for 2 min, then ramped at 10°C/min to 310°C, and held for 20 min. Inlet temperature: 310°C; Carrier gas: high‐purity He (99.999%), carrier gas flow rate 1.1 mL/min.

MS conditions: Ion source temperature 250°C, interface temperature 310°C, scan mass range 45–500 amu, solvent delay 2 min, ionization mode EI, electron energy 70 eV.

#### 2.2.7 Determination of 2,2‐Diphenyl‐1‐Picrylhydrazyl (DPPH) Radical Scavenging Rate

DPPH radical scavenging rate was determined according to the method of Jiang et al. (2024).

#### 2.2.8 Determination of Volatile Components

The method of Han et al. (2024) was adapted with modifications. One gram of oil sample was weighed into a 20 mL headspace vial, sealed with a silica‐coated PTFE membrane, and heated at 80°C in a metal bath for 25 min. After conditioning the extraction head, it was inserted into the headspace vial with the fiber tip extended and adsorbed for 30 min. Upon completion, the extraction head was removed and immediately inserted into the GC–MS inlet for desorption for 5 min.

GC Conditions: InertCap column (30.0 m × 0.25 mm, 0.25 μm); split injection, split ratio 20:1; temperature program: column oven initial temperature 40°C held for 2 min; ramp at 4°C/min to 180°C, held for 3 min; then ramp at 10°C/min to 250°C, held for 8 min; injector temperature 250°C; carrier gas: He, purge flow 3.0 mL/min.

MS Conditions: Full scan mode, electron impact ionization (70 eV), scan rate 0.5 scan/s, m/z range 33–500; ion source temperature 230°C; transfer line temperature 280°C; solvent delay time: 3 min.

Qualitative and semi‐quantitative analysis: The identification of volatile components was performed via computer‐aided compound information retrieval. During this process, compounds were searched simultaneously within the NIST spectral library alongside active peak identification. Compounds were selected as qualitative results only if they exhibited a similarity index greater than 80%. These results were then cross‐verified against the retention indices of corresponding compounds found in the literature. Finally, the relative content of the volatile components was calculated using the peak area normalization method.

#### 2.2.9 Relative Odor Activity Value (ROAV) Analysis

The ROAV is calculated using the following equation (Xu, Zhao, et al. 2023) to estimate the impact of flavor‐active compounds:

$${\text{ROAV}}_{i}=100\times \frac{{\mathrm{OAV}}_{i}}{{\mathrm{OAV}}_{\max}}$$

where ROAV_i_ represents the relative odor activity value of the volatile component; OAVmax denotes the maximum odor activity value among volatiles; OAVi is calculated via the equation OAVi = Ci/Ti, where Ci is the relative concentration, and Ti is the odor threshold. Components with ROAV ≥ 1 are characterized as key volatile constituents significantly contributing to the final aroma profile.

#### 2.2.10 Sensory Evaluation

Different CLCKO were evaluated using quantitative descriptive analysis. Ten trained panelists (4 males and 6 females) with an average age of 21 conducted sensory evaluations in a standardized sensory laboratory maintained at 27°C ± 2°C and 55% ± 3% relative humidity. Twenty milliliters of CLCKO were placed in test tubes, with samples randomly coded using Arabic numerals. Scoring was conducted for seven sensory attributes: lemony aroma, citrus aroma, oily aroma, herbaceous aroma, peppery aroma, floral‐fruity aroma, and burnt aroma. Intensity was rated on a 10‐point scale from 0 (no odor) to 9.0 (extremely strong odor), with increments of 1.0. The experiment was conducted in three rounds, and the sensory average score was calculated.

### 2.3 Data Statistics and Analysis

All experiments were performed in triplicate. Data are expressed as the mean ± standard deviation. Statistical analysis was conducted using one‐way analysis of variance (ANOVA) with IBM SPSS Statistics 26.0. FTIR spectral data were processed using OMNIC 9.2 software. OPLS‐DA was conducted using SIMCA 14.1 software to calculate the variable importance in projection (VIP). Infrared spectra, bar charts, radar charts, cluster heatmaps, and PCA plots were generated using Origin 2021 software.

## 3 Results and Analysis

### 3.1 FTIR Spectra of CLCKO After Different Pretreatments

**FIGURE 1:**
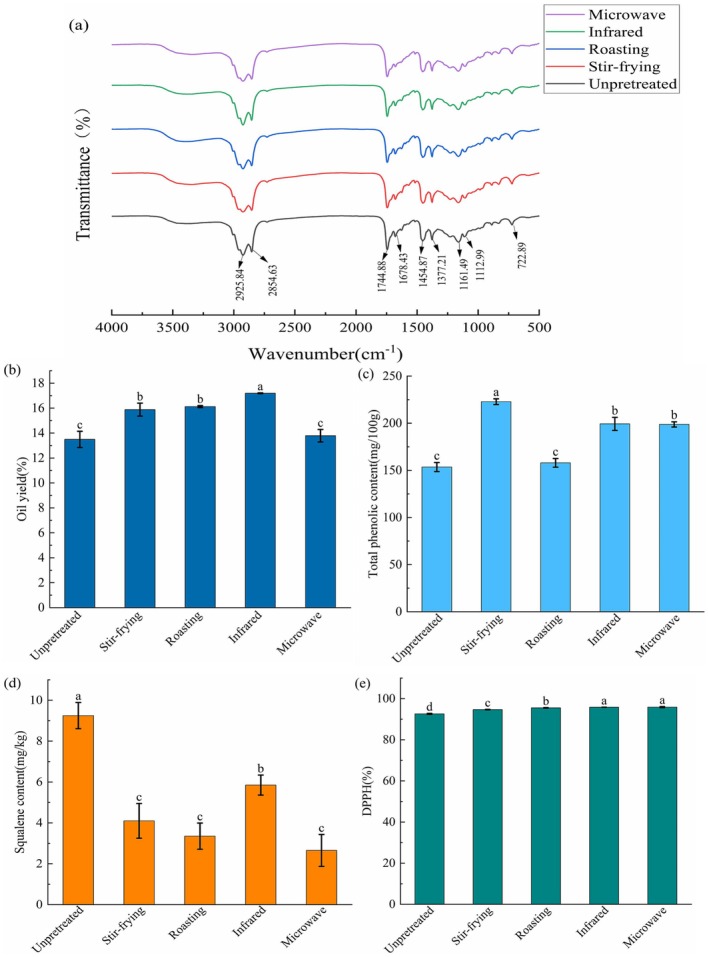
CLCKO with different pretreatment (a) Infrared spectrum; (b) Oil yield; (c) Total phenolic content; (d) Squalene concentration; (e) DPPH clearance rate.

The effects of different pretreatments on the FTIR spectra of CLCKO are shown in Figure 1a. The FTIR spectrum of CLCKO can be divided into five regions: (1) 2500–3500 cm^−1^; (2) 1650–1900 cm^−1^; (3) 1300–1500 cm^−1^; (4) 1100–1200 cm^−1^; (5) 700–900 cm^−1^. Two fatty acid OH group stretching vibration peaks appear at 2925.84 and 2854.63 cm^−1^, corresponding to the asymmetric and symmetric stretching vibrations of the C—H bond in the aliphatic CH_2_ functional group, respectively (Dessie et al. 2024). The absorption peak at 1744.88 cm^−1^ corresponds to the stretching vibration of the carbonyl (C=O) group, while the absorption peak at 1678.43 cm^−1^ corresponds to the stretching vibration of the di‐substituted cis C=C bond in unsaturated acyl groups. The 1454.87 and 1377.21 cm^−1^ bands are both C—H stretching peaks, corresponding to the bending vibration of C—H bonds in aliphatic CH_2_ and CH_3_ groups and the deformation vibration of C—H bonds in CH_2_ groups, respectively. The peak at 1161.49 cm^−1^ corresponds to the alkane backbone C—C vibration, and the peak at 1112.99 cm^−1^ corresponds to the C—O vibration. The absorption band at 722.89 cm^−1^ represents the out‐of‐plane rocking vibration of C—H bonds, indicating the presence of a chain structure with *n* ≥ 4 CH_2_ units. This finding is consistent with the FTIR analysis results reported by Lai et al. (2021) for LCKO extracted via aqueous enzymatic extraction, which likewise identified the aforementioned characteristic functional groups.

Compared with the unpretreated group, the overall signal patterns of CLCKO in different pretreatment groups were similar, indicating no change in functional group composition after pretreatment. However, the intensity of absorption peaks in the spectral region changed. The absorption peaks at 2925.84 and 2854.63 cm^−1^ exhibited lower intensities in the pretreated groups than in the control group. These peaks correlate with saturated fatty acid content (Kaur et al. 2019), which slightly decreased after pretreatment. The intensity of the 1744.88 cm^−1^ absorption peak also decreased after pretreatment. This aligns with findings by Wang et al. (2021), who observed reduced peak intensity at 1745 cm^−1^ in peony seeds after radiofrequency thermal pretreatment at different temperatures compared to unpretreated seeds. This change is speculated to be related to degradation or transformation of polysaccharide.

### 3.2 Effect of Different Pretreatment Technologies on the Oil Yield of CLCKO

As shown in Figure 1b, different pretreatment techniques can enhance the oil yield of CLCKO. The control group yielded 13.49% ± 0.65% oil. Pretreatment oil extraction rates increased by 0.14%–3.61%, likely due to thermal processing generating pressure through moisture evaporation in the kernels. This disrupts cellular structures and oil bodies, causing oil droplets to aggregate and release, significantly boosting extraction rates (Ma, Zheng, et al. 2024). This aligns with findings by Feng et al. (2025), who observed increased oil yields in camellia seeds after various pretreatments, including steaming, roasting, stir‐frying, microwave, and infrared heating. The oil yield in the microwave group was significantly lower than that in the roasting, infrared, and stir‐frying groups, and showed no significant difference from the unpretreated group. This phenomenon may be associated with microwave‐induced alterations in the physical properties of LCK. Wroniak et al. (2016) study on microwave pretreatment of rapeseed reported that excessively low moisture content in oilseeds increases material brittleness while concurrently reducing elasticity and ductility–thereby impeding effective oil extrusion and ultimately decreasing oil yield. Similarly, Huang et al. (2022) observed an analogous trend in perilla seeds: microwave pretreatment at 700 W for 10 min led to a reduction in oil yield. This decline is attributed to excessive microwave power and prolonged treatment duration, which promote excessive internal water evaporation, thereby diminishing material plasticity and adversely affecting oil extraction efficiency.

### 3.3 Effects of Different Pretreatment Technologies on the Physicochemical Properties of CLCKO

As shown in Table 1, the acid value of CLCKO ranged from 0.52 to 0.65 (mg KOH/g), while the peroxide value ranged from 0.07 to 0.10 (g/100 g). The infrared group exhibited higher acid values than the unpretreated group. This may be attributed to the polymerization reaction between volatile acids from phenolic acids generated during infrared pretreatment and free fatty acids. Over time, hydrolysis of phospholipids and triglycerides in LCK releases free fatty acids, leading to increased acid values (Suri et al. 2021). In contrast, acid values in the roasting, stir‐frying, and microwaved groups were lower than those in the control group. This aligns with Luo et al. (2018) findings that acid value decreased in pressed walnut oil after walnuts were roasted at 150°C for 1 h. The roasting group exhibited a higher peroxide value than the unpretreated group, while the stir‐frying and microwave groups showed lower values. This discrepancy may arise because during lipid oxidation, the formation rate of peroxides from secondary oxidation reactions (involving aldehydes, ketones, and epoxides) is lower than their decomposition rate, leading to decreased peroxide values, whereas the opposite occurs when the formation rate exceeds the decomposition rate (Cao et al. 2024).

**TABLE 1:** Physical and chemical properties of CLCKO with different pretreatment.

	Unpretreated	Stir‐frying	Roasting	Infrared	Microwave
Acid value (mg/g)	0.61 ± 0.06^a^	0.57 ± 0.11^a^	0.55 ± 0.08^a^	0.65 ± 0.03^a^	0.52 ± 0.04^a^
Peroxide value (g/100 g)	0.09 ± 0.01^a^	0.07 ± 0.01^a^	0.10 ± 0.01^a^	0.09 ± 0.01^a^	0.08 ± 0.01^a^
Anisidine value (%)	9.42 ± 0.08^d^	10.71 ± 0.13^c^	11.73 ± 0.04^a^	11.37 ± 0.13^b^	6.45 ± 0.13^e^

*Note:* Different lowercase letters (a–e) in the chart indicate significant differences (*p* < 0.05), while the same lowercase letters indicate insignificant differences (*p* > 0.05).

The anise amine value measures the concentration of aldehydes produced during hydrogen peroxide decomposition, indicating the degree of secondary oxidation. As shown in Table 1, the anisidine value of CLCKO ranged from 6.45% to 11.73%. Compared to the unpretreated group, the anisidine values of the stir‐frying, roasting, and infrared‐treated groups all significantly increased by 1.29%, 2.31%, and 1.95%, respectively. This phenomenon may arise from synergistic interactions between the Maillard reaction and lipid oxidation under high‐temperature conditions. Specifically, the Maillard reaction depletes reducing agents in the system, weakening its protective effect against lipid oxidation and enhancing the conversion rate of primary oxidation products into secondary oxidation products. This facilitates the formation of secondary oxidation products such as ketones, aldehydes, and quinones in CLCKO (Hashemi et al. 2016). Conversely, the decrease in anise amine value in the microwave group (Hashemi et al. 2016) may stem from the microwave's rapid and short‐duration nature of heating, which minimizes oxidative stress on lipids during thermal processing. Microwaves may also promote further decomposition or conversion of aldehydes, thereby reducing the accumulation of secondary oxidation products and preventing the formation of substantial aldehyde compounds (Kaseke et al. 2020).

### 3.4 Effects of Different Pretreatment Technologies on Fatty Acids in CLCKO

The fatty acid composition reflects the quality of oils and fats, influencing their nutritional value and oxidative stability. As shown in Table 2, different pretreatment techniques have little effect on the fatty acid composition of CLCKO. This oil primarily consists of linoleic acid (27.13%–30.37%), lauric acid (24.51%–27.03%), oleic acid (17.57%–19.32%), palmitic acid (15.00%–15.30%), capric acid (4.58%–4.74%), linolenic acid (2.43%–3.33%), myristic acid (1.27%–1.49%), palmitoleic acid (1.44%–1.65%), stearic acid (0.85%–0.97%), and tetradecenic acid (0.35%–0.41%). Among these, linoleic acid and lauric acid are the predominant fatty acids, accounting for over 50% of the total content, followed by oleic acid and palmitic acid. This composition is largely consistent with the findings of Yang, Yang, et al. (2024) regarding the major fatty acids in LCKO. However, some previous studies have indicated a higher lauric acid content in LCKO. For instance, Li et al. (2016) used petroleum ether reflux extraction on kernel oil from Xiangxi, Hunan, yielding a lauric acid content of 49.53%. Zhuang (2019) extracted LCKO from Qingyuan using methods like pressing and solid–liquid extraction, with lauric acid content exceeding 50% in both cases. In contrast, lauric acid ranked second in this study. This discrepancy may stem from two factors: firstly, variations in *L. cubeba's* origin or harvesting season can influence lauric acid content (Li et al. 2016); secondly, elevated pretreatment temperatures may cause fluctuations in lauric acid accumulation (Lv et al. 2017). This indicates that fatty acid composition ratios may be affected by geographical origin, genetic variants, or extraction conditions (Jiang et al. 2024).

**TABLE 2:** Effect of different pretreatment techniques on fatty acid composition of CLCKO.

Fatty acids	Name	Unpretreated	Stir‐frying	Roasting	Infrared	Microwave
C10:0	Decanoic acid	4.74 ± 0.04^a^	4.58 ± 0.12^a^	4.67 ± 0.03^a^	4.67 ± 0.07^a^	4.68 ± 0.08^a^
C12:0	Lauric acid	27.03 ± 0.14^a^	24.51 ± 0.98^b^	24.96 ± 0.29^b^	25.45 ± 0.10^b^	25.33 ± 0.05^b^
C14:0	Myristic acid	1.37 ± 0.00^b^	1.49 ± 0.00^a^	1.41 ± 0.01^b^	1.42 ± 0.06^ab^	1.27 ± 0.01^c^
C14:1	Myristelaidic acid	0.35 ± 0.01^a^	0.37 ± 0.05^a^	0.41 ± 0.02^a^	0.41 ± 0.03^a^	0.40 ± 0.00^a^
C16:0	Palmitic acid	15.29 ± 0.09^a^	15.21 ± 0.23^a^	15.30 ± 0.35^a^	15.00 ± 0.13^a^	15.13 ± 0.04^a^
C16:1	Palmitoleic acid	1.49 ± 0.04^b^	1.60 ± 0.03^a^	1.65 ± 0.01^a^	1.64 ± 0.05^a^	1.44 ± 0.06^b^
C18:0	Stearic acid	0.85 ± 0.07^a^	0.97 ± 0.07^a^	0.93 ± 0.00^a^	0.92 ± 0.05^a^	0.94 ± 0.02^a^
C18:1	Oleic acid	19.32 ± 0.17^a^	17.57 ± 0.35^c^	18.45 ± 0.48^abc^	18.51 ± 0.51^ab^	17.9 ± 0.13^bc^
C18:2	Linoleic acid	27.13 ± 0.12^b^	30.37 ± 1.17^a^	29.14 ± 0.48^a^	29.02 ± 0.52^a^	29.6 ± 0.22^a^
C18:3	Linolenic acid	2.43 ± 0.10^c^	3.33 ± 0.18^a^	3.08 ± 0.12^ab^	2.96 ± 0.13^b^	3.31 ± 0.01^a^
SFA	Saturated fatty acids	49.28 ± 0.18^a^	46.76 ± 0.93^b^	47.27 ± 0.08^b^	47.46 ± 0.15^b^	47.35 ± 0.13^b^
UFA	Unsaturated fatty acid	50.72 ± 0.18^b^	53.24 ± 0.93^a^	52.73 ± 0.08^a^	52.54 ± 0.15^a^	52.65 ± 0.13^a^

*Note:* Different lowercase letters (a–c) in the chart indicate significant differences (*p* < 0.05), while the same lowercase letters indicate insignificant differences (*p* > 0.05).

In this study, the saturated fatty acid content of CLCKO ranged from 46.76% to 49.28%, while the unsaturated fatty acid content ranged from 50.72% to 53.24%. Compared with the unpretreated group, the saturated fatty acid content decreased by 2.56%, 2.01%, 1.82%, and 1.93% in the stir‐frying, roasting, infrared, and microwave groups, respectively. The unsaturated fatty acid content increased by 2.52%, 2.01%, 1.82%, and 1.93%, respectively, and the differences in fatty acid content between different pretreatments were relatively small. This may also be attributed to heat‐induced formation of high‐molecular‐weight compounds in oils and fats—some of which may persist during fatty acid methylation analysis. This potentially led to a relative underestimation of saturated fatty acid content. Concurrently, thermal treatment may facilitate the transformation of certain polar lipid components, resulting in a slight relative increase in unsaturated fatty acid proportions. This finding is consistent with the results reported by Xiong et al. (2025), who observed a decrease in saturated fatty acid content and an increase in unsaturated fatty acid content in pretreated camellia seed oil. It also aligns with the infrared spectroscopy measurements shown in Figure 1a.

### 3.5 Effect of Different Pretreatment Technologies on Total Phenolic Content in CLCKO

The effects of different pretreatment techniques on the total phenolic content of CLCKO are shown in Figure 1c. Different pretreatment techniques can all increase the total phenolic content of CLCKO, increased by 4.43%–69.36%. This is consistent with the findings of Jiang et al. (2024) that various pretreatments (microwave, infrared, roasting, and enzymatic methods) can enhance the total phenolic content of cold‐pressed rapeseed oil.

As shown in Figure 1c, the stir‐frying group exhibited the highest total phenolic content. This difference may stem from variations in heat transfer characteristics and mechanisms among different thermal treatments, leading to distinct equilibria in the release and degradation of phenolic compounds. Stir‐frying heat treatment employs a combination of heat conduction, continuous gentle stirring, and sustained heating. This process disrupts the internal structure of the kernel, denatures phenolic compound proteins, and subsequently converts bound phenols into free or easily precipitable forms (Yang et al. 2015). The roasting group exhibited the lowest total phenolic content, likely due to oxidative degradation caused by prolonged exposure to high temperatures, which interferes with the stability of phenolic compounds. This finding aligns with the results reported by Gao et al. (2019), who observed a decrease in total phenolic content in walnut oil during prolonged roasting due to thermal degradation of phenolic compounds at elevated temperatures. Microwave pretreatment generates heat within the material through the interaction between the microwave electromagnetic field and polar molecules in the material (Huang et al. 2022). Infrared pretreatment utilizes infrared radiation to induce molecular vibration and heat generation within the material, thereby achieving the heating process (Huang et al. 2023). Both methods yielded lower total phenolic content than the stir‐frying group, likely due to differing combinations of dehydration effects, thermal action patterns, and processing times (Ahmed et al. 2021).

### 3.6 Effect of Different Pretreatment Technologies on the Squalene Content in CLCKO

Squalene is a triterpene compound with an isoprene structure. It not only possesses strong antioxidant properties but also reduces cholesterol synthesis, enhances immune system function, inhibits tumor cell proliferation, and mitigates the adverse effects of external toxic substances on the body (Song et al. 2020). This study measured squalene content in CLCKO ranging from 2.65 to 9.25 mg/kg (Figure 1d). Compared to the unpretreated group, squalene concentrations decreased in all pretreatment groups. This phenomenon may result from several factors: First, as a precursor in the epoxidase metabolic pathway, squalene's final metabolic process may not have been fully completed (Dąbrowski et al. 2024). Secondly, after extracting LCEO via steam distillation, the distillation residue underwent pretreatment and oil extraction through pressing. In this process, the multi‐step sequence of steam distillation, residue pretreatment, and oil extraction by pressing, as compared to direct pressing (Li, Yang, et al. 2024), involved multiple thermal operations. These stages may have accelerated the thermal decomposition of squalene, potentially leading to the observed reduction in squalene content (Yang et al. 2025).

### 3.7 Effect of Different Pretreatment Techniques on DPPH Radical Scavenging Rate of CLCKO

The antioxidant capacity of CLCKO is a key factor in evaluating its sensory and nutritional qualities. Oxidative degradation of oils leads to significant deterioration in oil quality. We assessed its antioxidant capacity by measuring its DPPH radical scavenging rate. As shown in Figure 1e, the DPPH radical scavenging capacity of CLCKO ranged from 92.59% to 95.88%, and its radical scavenging ability was enhanced after different pretreatments. Overall, the infrared and microwave pretreatment group exhibited stronger antioxidant capacity. This may be attributed to microwave pretreatment disrupting the cellular structure of oilseeds, promoting the release of phenolic compounds into the oil, and is also directly correlated with its higher total phenolic content (Li, Zhu, et al. 2025). However, although the stir‐frying sample exhibited the highest total phenolic content, its DPPH radical scavenging activity was comparatively lower—highlighting the dual and opposing effects of thermal processing on the antioxidant functionality of phenolic compounds. Specifically, elevated temperatures facilitate the release of bound phenolics, thereby increasing measurable total phenolic content (Harivaindaran et al. 2023). On the other hand, prolonged or intense heating may induce structural modifications—including oxidation, polymerization, or isomerization—of certain phenolic compounds, potentially yielding derivatives with reduced reactivity toward DPPH radicals or altered redox behavior, which collectively contributes to the observed decline in DPPH scavenging capacity. This finding aligns with the results reported by Lutterodt et al. (2010), who observed that black seed oil exhibits high total phenolic content but low DPPH values. Conversely, the DPPH radical scavenging capacity of the roasting pretreatment was lower, likely due to its inherently lower total phenolic content.

### 3.8 Analysis of Volatile Aroma Compounds in CLCKO

Flavor is one of the important indicators for measuring the overall quality of CLCKO. GC–MS results for CLCKO extracted after different pretreatments (stir‐frying, roasting, infrared, microwave) are shown in Table 3. The results revealed that a total of 43 volatile compounds were identified across the differently pretreated samples, predominantly comprising terpenes (26 compounds), alcohols (5), aldehydes (4), ketones (3), esters (1), and other oxygenated compounds (4). The types and relative abundances of these compounds varied to a certain extent depending on the pretreatment method employed. This variation was closely associated with the synergistic effects of multiple thermal reactions—including the Maillard reaction, Strecker degradation, caramelization, and lipid thermal oxidation and degradation—occurring during thermal processing (Liu et al. 2024).

**TABLE 3:** Volatile flavor components of CLCKO.

	CAS NO	Compound name	Odor descriptors (Qiao et al. 2008; Liu et al. 2012; Rao et al. 2025)	Unpretreated	Stir‐frying	Roasting	Infrared	Microwave
1	6709‐39‐3	Geraniolene	—	—	—	—	—	0.14 ± 0.03
2	508‐32‐7	Cyclene	—	0.02 ± 0.00	0.02 ± 0.01	0.04 ± 0.01	0.03 ± 0.00	0.04 ± 0.01
3	2867‐5‐2	α‐Myrcene	Woody, greenish, grassy	0.13 ± 0.01	0.19 ± 0.02	0.23 ± 0.01	0.33 ± 0.02	0.42 ± 0.01
4	3779‐61‐1	(E)‐β‐Ocimene	Sweet, herbal	6.85 ± 0.09	7.55 ± 0.58	7.91 ± 0.08	7.95 ± 0.28	8.02 ± 0.17
5	79‐92‐5	(+)‐Camphene	—	1.43 ± 0.03	1.51 ± 013	1.78 ± 0.06	1.78 ± 0.10	1.78 ± 0.03
6	3387‐41‐5	Sabinene	Woody, spicy and Turpentine	1.03 ± 0.05	0.75 ± 0.06	0.69 ± 0.13	0.61 ± 0.02	0.35 ± 0.06
7	127‐91‐3	β‐pinene	Piney	3.17 ± 0.03	3.68 ± 0.07	3.68 ± 0.14	3.87 ± 0.18	3.80 ± 0.03
8	123‐35‐3	Myrcene	Peppery, spicy	7.62 ± 0.11	7.92 ± 0.14	8.81 ± 0.18	9.25 ± 0.65	9.33 ± 0.08
9	99‐83‐2	α‐phellandrene	Citrus, lime, fresh green	—	0.05 ± 0.01	0.04 ± 0.00	0.06 ± 0.00	0.20 ± 0.01
10	554‐61‐0	2‐Carene	—	0.10 ± 0.01	0.15 ± 0.01	0.19 ± 0.01	0.23 ± 0.04	0.42 ± 0.04
11	138‐86‐3	Limonene	Lemon, Minty, Orange	44.54 ± 0.73	46.48 ± 0.45	45.97 ± 0.62	46.20 ± 1.99	46.28 ± 1.87
12	502‐99‐8	Alpha‐Ocimene	Citrus, grassy, woody	—	0.02 ± 0.00	0.02 ± 0.00	0.02 ± 0.00	0.09 ± 0.01
13	99‐85‐4	γ‐terpinene	Sweet, Citrusy	0.24 ± 0.03	0.40 ± 0.03	0.50 ± 0.02	0.50 ± 0.20	0.79 ± 0.22
14	586‐62‐9	Terpinolene	Green	0.20 ± 0.02	0.22 ± 0.03	0.29 ± 0.01	0.30 ± 0.04	0.49 ± 0.02
15	3856‐25‐5	α‐Copaene	Woody, spicy, honey	1.66 ± 0.12	1.91 ± 0.04	1.88 ± 0.10	1.80 ± 0.34	1.91 ± 0.10
16	33880‐83‐0	Beta‐elemene	Herbal, waxy, fresh	0.47 ± 0.06	0.74 ± 0.08	0.95 ± 0.01	0.81 ± 0.08	0.64 ± 0.30
17	87‐44‐5	(−)‐β‐caryophyllene	Sweet, Woody, Spicy	6.37 ± 0.35	7.16 ± 0.04	6.89 ± 0.14	6.59 ± 1.07	7.29 ± 0.29
18	17699‐05‐7	alpha‐Bergamotene	—	0.18 ± 0.01	0.20 ± 0.01	0.21 ± 0.01	0.18 ± 0.01	0.20 ± 0.00
19	28973‐97‐9	(Z)‐beta‐farnesene	Citrus, green	2.99 ± 0.06	3.51 ± 0.08	3.21 ± 0.14	3.11 ± 0.69	0.08 ± 0.01
20	473‐13‐2	α‐selinene		0.05 ± 0.00	0.07 ± 0.00	0.06 ± 0.00	0.06 ± 0.01	0.04 ± 0.00
21	29873‐99‐2	γ‐Elemene	Green, woody, oily	0.47 ± 0.01	0.52 ± 0.01	0.46 ± 0.01	0.45 ± 0.12	0.35 ± 0.01
22	6753‐98‐6	Alpha‐caryophyllene	Woody	0.04 ± 0.01	0.05 ± 0.00	0.05 ± 0.01	0.04 ± 0.01	0.05 ± 0.00
23	495‐61‐4	β‐Bisabolene	Balsamic, woody	0.68 ± 0.04	0.90 ± 0.00	0.75 ± 0.02	0.78 ± 0.19	0.90 ± 0.03
24	483‐76‐1	(+)‐δ‐cadinene	Thyme, herbal, woody	0.27 ± 0.00	0.35 ± 0.01	0.31 ± 0.01	0.31 ± 0.08	0.37 ± 0.01
25	20307‐83‐9	beta‐sesquiphellandrene	Herbal, fruity, woody	0.15 ± 0.00	0.18 ± 0.01	0.16 ± 0.01	0.15 ± 0.02	0.16 ± 0.00
26	502‐61‐4	Farnesene	Woody, green, floral	—	0.04 ± 0.00	0.02 ± 0.00	0.04 ± 0.01	0.16 ± 0.01
27	78‐70‐6	Linalool	Fruity	0.64 ± 0.05	0.62 ± 0.04	0.70 ± 0.05	0.69 ± 0.01	0.70 ± 0.07
28	562‐74‐3	4‐Terpene alcohol	Pine‐like, sweet, green	0.06 ± 0.01	0.10 ± 0.01	0.08 ± 0.00	0.10 ± 0.01	0.09 ± 0.02
29	473‐67‐6	Verbenol	Herbal pine	0.09 ± 0.00	0.06 ± 0.00	0.03 ± 0.00	0.06 ± 0.00	0.10 ± 0.01
30	98‐55‐5	α‐Terpineol	Lemon, Piney, Minty	0.17 ± 0.00	0.18 ± 0.00	0.17 ± 0.02	0.17 ± 0.01	0.20 ± 0.02
31	106‐24‐1	Geraniol	Floral, Lemon, Minty	0.18 ± 0.01	0.28 ± 0.06	0.31 ± 0.04	0.31 ± 0.01	0.37 ± 0.02
32	97‐96‐1	2‐Ethyl butyraldehyde	Green	0.03 ± 0.01	0.03 ± 0.01	0.04 ± 0.00	0.06 ± 0.01	0.07 ± 0.01
33	106‐23‐0	Citronellal	Floral, green, rosy and citrus lemon	0.27 ± 0.02	0.22 ± 0.00	0.24 ± 0.00	0.25 ± 0.02	0.21 ± 0.02
34	106‐26‐3	Neral	Rose	6.57 ± 0.24	4.27 ± 0.04	4.25 ± 0.14	4.06 ± 0.33	3.01 ± 0.23
35	141‐27‐5	Geranial	Floral, Citrusy	11.48 ± 0.40	7.24 ± 0.26	7.33 ± 0.21	6.99 ± 0.28	5.37 ± 0.28
36	1193‐18‐6	Methylcyclohexenone	Nutty	—	—	—	—	0.04 ± 0.01
37	80‐57‐9	(S)‐verbenone	Camphoreous	0.18 ± 0.01	0.25 ± 0.02	0.29 ± 0.03	0.29 ± 0.01	0.46 ± 0.04
38	18309‐32‐5	Verbenone	Camphoreous	0.12 ± 0.04	0.10 ± 0.01	0.02 ± 0.02	0.02 ± 0.00	3.21 ± 0.20
39	80‐26‐2	Acetic acid pine oil alcohol	Woody, citrus, spicy, floral, grapefruit and seedy	0.39 ± 0.04	0.37 ± 0.05	0.38 ± 0.04	0.33 ± 0.03	0.39 ± 0.00
40	539‐52‐6	Perillene	Woody	—	0.02 ± 0.04	0.03 ± 0.01	0.03 ± 0.00	0.03 ± 0.00
41	19888‐34‐7	Humulene epoxide II	—	0.06 ± 0.00	0.18 ± 0.01	0.07 ± 0.01	0.10 ± 0.04	0.25 ± 0.01
42	1139‐30‐6	(−)‐Caryophyllene oxide	Woody	1.19 ± 0.04	1.51 ± 0.01	1.05 ± 0.03	1.17 ± 0.33	1.24 ± 0.01
43	34995‐77‐2	(E)‐linalool oxide (furanoid)	Floral	—	—	—	—	0.06 ± 0.01

*Note:* The aroma descriptions of substances are obtained from the query database http://www.thegoodscentscompany.com/ and references (Qiao et al. 2008; Liu et al. 2012; Rao et al. 2025). “—” means not detected.

Terpenoid compounds represent the most abundant and chemically diverse class of small‐molecule natural products, primarily generated through the terpenoid metabolic pathway (Yi et al. 2016). They constitute the major volatile components of CLCKO, accounting for the highest relative content at 78.61%–85.39%. A total of 22 terpenoid compounds were identified in the unpretreated group, 25 in the roasting group, stir‐frying group and infrared group, and 26 in the microwave group. In contrast, the compounds of geraniolene, α‐phellandrene, alpha‐ocimene, perillene and farnesene, only detected in the pretreatment group, jointly endowed with flavor characteristics such as the orange, green grass, woody, and floral aromas of CLCKO. Among the identified terpenes, limonene and myrcene exhibited the highest relative abundances, jointly accounting for over 50% of the total terpenoid content—thereby imparting characteristic lemon and peppery aroma notes to the oil. Compared with the unpretreated group, the pretreatment group demonstrated a statistically significant increase in the relative abundance of several key terpenoids, including limonene, myrcene, (E)‐β‐Ocimene, β‐pinene, (−)‐β‐caryophyllene, β‐bisabolene and α‐copaene. Consequently, sensory attributes—including citrus, pepper, sweet, herbal, woody, balsamic, and camphoraceous aromas—were notably enhanced. This may result from insufficient extraction of terpenoids into the oil in the control group due to the absence of thermal pretreatment (Ni et al. 2021).

Aldehydes represent the second most abundant class of volatile compounds in CLCKO, following terpenes, with concentrations ranging from 8.66% to 18.34%. Owing to their low odor thresholds, aldehydes significantly contribute to aroma intensity and serve as key flavor enhancers; they are predominantly formed via oxidative degradation of unsaturated fatty acids (Stübner and Steinhaus 2023). Four aldehyde species were identified in CLCKO: 2‐ethylbutanal, citronellal, neral, and geranial. Notably, citral—which comprises the geometric isomers neral and geranial—accounted for over 90% of the total aldehyde content. Following thermal pretreatment, the overall aldehyde concentration in CLCKO decreased markedly. This decline is likely attributable to carbonyl–amine condensation reactions involving highly reactive aldehydes, followed by cyclization and dehydration to yield heterocyclic compounds (e.g., Strecker aldehydes, pyrazines, or furans) under elevated temperatures (Zeng et al. 2025). Among them, the aldehyde compound content in the microwave pretreatment group was the lowest, indicating that this treatment method caused the most significant loss of lemon aroma, which is consistent with its lowest anisidine value.

Ketone compounds primarily form through amino acid decomposition and thermal oxidation degradation of polyunsaturated fatty acids (Xu, Bi, et al. 2023). Possessing extremely low thresholds and typically exhibiting faint odors, they significantly influence the overall aroma of heated oils. Only three ketone compounds—3‐Methyl‐2‐cyclohexen‐1‐one, (S)‐verbenone, and verbenone—were detected in CLCKO. Compared to the unpretreated group, the microwave group showed a significant increase in verbenone content (3.21%), enhancing its camphoreous aroma, while other pretreatment groups exhibited decreased levels and weakened camphor notes.

Volatile alcohols primarily originate from fat oxidation or aldehyde reduction (Xu, Bi, et al. 2023). In general, due to the low alcohol content (1.13%–1.44%), the impact on the flavor of CLCKO can be ignored. Five types of alcohols were detected in the CLCKO, among which terpinen‐4‐ol had a lower content, higher sensory threshold, and lower contribution to the overall flavor. The sensory threshold of verbenol was not retrieved and its content is low, indicating that its contribution to the overall flavor needs to be explored. Linalool, α‐terpineol, and geraniol have lower odor thresholds and are endowed with fruity and minty flavors, enriching the flavor of CLCKO. In addition, compared with unpretreated, except for a slight decrease in linalool content in stir‐frying. The linalool content in all other pretreatment groups increased. All pretreatment groups showed an increase in geraniol content, indicating a stronger fruity and floral flavor after pretreatment.

**FIGURE 2:**
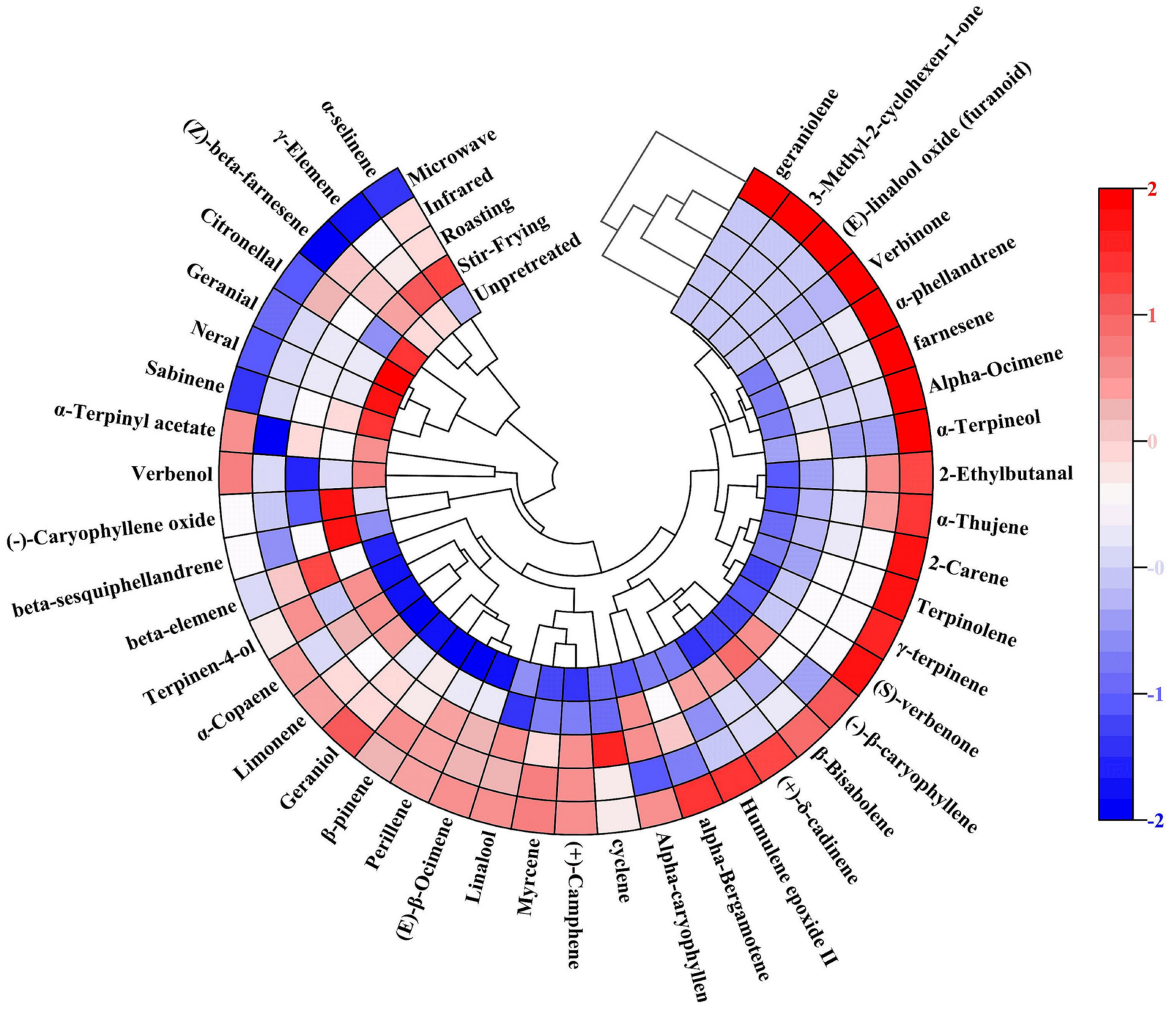
Cluster thermogram of volatile flavor compounds in CLCKO.

To systematically characterize the differential distribution patterns of volatile flavor compounds in CLCKO under various pretreatment conditions, this study employed hierarchical cluster analysis coupled with heatmap visualization (Figure 2). Clear separation was observed among the volatile profiles of the unpretreated group and all pretreatment groups—stir‐frying, roasting, infrared, and microwave groups—demonstrating that thermal pretreatments markedly modulate the composition of volatile flavor compounds. Cluster analysis further revealed that the microwave group exhibited the greatest divergence from the unpretreated group, followed by the infrared and roasting groups. Notably, the stir‐frying group displayed the most subtle alterations, suggesting a comparatively milder impact on flavor compound regulation. These findings collectively indicate that the type and intensity of thermal processing exert distinct and quantifiable influences on the volatile flavor profile of CLCKO.

### 3.9 OPLS‐DA‐Based Multivariate Analysis of Volatile Flavor Compounds in CLCKO

**FIGURE 3:**
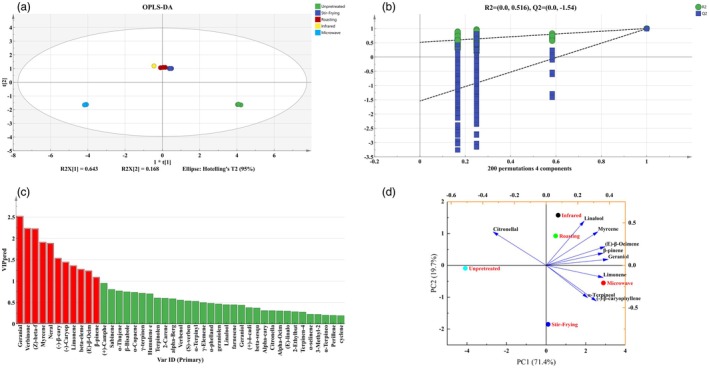
(a) OPLS‐DA score chart, (b) The graph is cross‐verified by 200 permutation tests, (c) VIP score, red corresponds to VIP ≥ 1 compounds and green indicates VIP < 1 compounds, (d) PCA biplot of key aroma compounds.

OPLS‐DA, a supervised multivariate statistical method based on partial least squares regression (Chen et al. 2024), was employed to evaluate intergroup differences in volatile flavor compounds among CLCKO samples subjected to distinct pretreatment protocols. In contrast to unsupervised principal component analysis (PCA), OPLS‐DA maximizes class separation while filtering out orthogonal variation unrelated to group discrimination, thereby enabling robust identification of characteristic volatiles underpinning aroma differentiation. As shown in the OPLS‐DA score plot (Figure 3a), the five sample groups exhibited complete spatial segregation, with excellent model fit and predictive capability (*R*_*X*_^2^ = 0.993, *R*_*Y*_^2^ = 0.975, *Q* ^2^ = 0.874). The *R* ^2^ and *Q* ^2^ values exceeding 0.5 collectively validate the model'sexplanatory power and cross‐validated robustness. Relative to the unpretreated, all pretreated samples occupied distinct positions in the two‐dimensional score space, confirming that pretreatment significantly alters the volatile profile—and consequently the aroma characteristics—of CLCKO. This highlights pretreatment as a critical process parameter governing both the formation of flavor compounds and compositional divergence. Along the predictive x‐axis, roasting, infrared, and stir‐frying displayed comparable intergroup distances, whereas microwave exhibited the greatest uclidean distance from the unpretreated, suggesting its most pronounced impact on aroma modulation. To rigorously assess model validity and guard against overfitting, a permutation test (*n* = 200) was performed. The resulting *Q* ^2^ intercept (−1.54) fell well below zero (*R* ^2^ intercept = 0.516), confirming the absence of overfitting and affirming the statistical soundness of the model (Figure 3b). Variable Importance in Projection (VIP) scores were further used to quantify each volatile compound's contribution to class discrimination; compounds with VIP ≥ 1 are conventionally regarded as key discriminators with high classification relevance (Giannetti et al. 2017) (Figure 3c). To enhance confidence in differential identification, VIP ≥ 1 was combined with nonparametric Kruskal–Wallis testing (*p* < 0.05) for intergroup significance. This dual‐criteria approach yielded eight statistically robust and biologically relevant differential aroma compounds (Table 4), which collectively serve as chemometric markers capable of reliably distinguishing among the different pretreatment methods.

**TABLE 4:** The differential VOCs of CLCKO under different heat pretreatments based on VIP ≥ 1 and *p* < 0.05.

NO.	Compound name	VIP	*p*	Pretreatment methods
37	Geranial	2.524	0.017	Stir‐frying, Roasting, Microwave, Infrared
20	Verbenone	2.242	0.015	Stir‐frying, Roasting, Microwave, Infrared
40	(Z)‐beta‐farnesene	2.234	0.033	Stir‐frying, Roasting, Microwave, Infrared
36	Myrcene	1.916	0.016	Stir‐frying, Roasting, Microwave, Infrared
8	Neral	1.891	0.023	Stir‐frying, Roasting, Microwave, Infrared
7	(−)‐Caryophyllene oxide	1.451	0.034	Stir‐frying, Roasting, Microwave, Infrared
42	Beta‐elemene	1.284	0.036	Stir‐frying, Roasting, Microwave, Infrared
17	β‐pinene	1.096	0.023	Stir‐frying, Roasting, Microwave, Infrared

### 3.10 Effect of Pretreatment on ROAV of Key Aroma Compounds in CLCKO

To evaluate the impact of different preprocessing methods on the flavor contribution of CLCKO, ROAV were employed to quantitatively analyze each volatile compound. The larger the ROAV value, the more significant the contribution of the compound to the overall flavor. Generally, compounds with ROAV ≥ 1 are considered as key aroma components (KACs), whereas those with 0.1 ≤ ROAV < 1 are regarded as aroma‐modulating compounds that fine‐tune the overall olfactory profile (Ma, Wang, et al. 2024). As shown in Table 5, among the 17 major aroma‐active compounds identified in CLCKO, nine—including (E)‐β‐ocimene, β‐pinene, myrcene, limonene, (−)‐β‐caryophyllene, linalool, α‐terpineol, geraniol, and citronellal—exhibit ROAVs ≥ 1, collectively constituting the core aromatic backbone of CLCKO. Notably, (−)‐β‐caryophyllene displayed a consistently maximal ROAV of 100.00 across all samples, establishing it as the predominant aroma contributor and imparting a pronounced spicy–woody character. β‐Pinene exhibited high ROAVs ranging from 53.14 to 62.69, conferring a distinct pine‐resinous note; likewise, citronellal showed ROAVs between 41.20 and 60.51, contributing robust floral, lemony, and citrus‐like aromas. Additionally, eight compounds—including sabinene, γ‐terpinene, and terpinolene—with ROAVs in the range of 0.1–0.99 function as aroma modulators, subtly shaping the overall olfactory balance of CLCKO. Although ROAVs of individual KACs varied slightly under different pretreatment conditions, (−)‐β‐caryophyllene, β‐pinene, and citronellal consistently remained dominant. This suggests that thermal pretreatment exerts minimal influence on the fundamental aroma character of LCKO, primarily modulating only the relative abundance and intensity of certain volatile constituents.

**TABLE 5:** The key flavor substance ROAV of CLCKO with different pretreatment.

Serial number	Volatile flavor compounds	Ti (mg/kg)	ROAV				
			Unpretreated	Stir‐Frying	Roasting	Infrared	Microwave
1	(E)‐β‐Ocimene	0.034^a^	20.26	19.83	21.61	22.73	20.73
2	Sabinene	0.98^a^	0.10	0.07	0.06	0.06	0.03
3	β‐pinene	0.006^b^	53.14	54.74	56.97	62.69	55.60
4	Myrcene	0.0445^a^	17.18	15.89	18.38	20.20	18.36
5	α‐phellandrene	0.036^a^	—	0.11	0.10	0.16	0.50
6	Limonene	0.25^a^	17.96	16.61	17.08	17.96	16.31
7	Alpha‐Ocimene	0.034^a^	—	0.05	0.05	0.06	0.21
8	γ‐terpinene	1.49^a^	0.02	0.02	0.03	0.03	0.05
9	Terpinolene	0.227^a^	0.09	0.09	0.12	0.13	0.19
10	(−)‐β‐caryophyllene	0.0064^b^	100.00	100.00	100.00	100.00	100.00
11	Linalool	0.037^c^	1.72	1.49	1.74	1.80	1.65
12	Terpinen‐4‐ol	1.2^a^	—	0.01	0.01	0.01	0.01
13	α‐Terpineol	0.01^b^	1.71	1.59	1.53	1.65	1.72
14	Geraniol	0.001^b^	17.58	24.93	28.33	30.13	32.16
15	Citronellal	0.00044^b^	60.51	43.97	50.67	54.12	41.20
16	α‐Terpinyl acetate	2.5^a^	0.02	0.01	0.01	0.01	0.01
17	(E)‐linalool oxide (furanoid)	0.32^a^	—	—	—	—	0.02

*Note:* —, not detected. Ti: odor threshold, the data from (a) Sun et al. (2020); (b) Ni et al. (2021); (c) Van Gemert (2011).

### 3.11 Principal Component Analysis of Key Aroma Compounds in CLCKO

To clarify the correlation between aroma compounds and different pretreatment methods, principal component analysis (PCA) was conducted on 9 key aroma compounds (KACs). The first two principal components—PC1 and PC2—accounted for 71.4% and 19.7% of the total variance, respectively, yielding a cumulative contribution rate of 91.1%. This high cumulative variance indicates that the PCA model effectively captures the overall variation in aroma profiles across samples from different treatment groups, thereby highlighting the substantial influence of pretreatment methods on flavor characteristics. In the PCA biplot (Figure 3d), sample points represent the score distribution of differently pretreated samples, while blue arrows denote the loading vectors of the aroma compounds. The spatial proximity between an arrow (compound) and a sample point reflects their correlation: the shorter the distance, the stronger the association. There were significant differences in the characteristics of aroma components between different pretreatment groups: the sample points in the unpretreated group were located in the negative region of PC1 and exhibited strong positive correlation with citronellal. Infrared and roasting samples were located in the first quadrant and were predominantly characterized by linalool, myrcene, (E)‐β‐ocimene, β‐pinene, and geraniol. The samples treated with microwave tended toward the positive direction of PC1 and the negative direction of PC2, showing pronounced correlations with limonene, α‐terpineol, and (−)‐β‐caryophyllene. Stir‐frying samples were positioned along the negative half‐axis of PC2 and also showed close associations with α‐terpineol and (−)‐β‐caryophyllene. Collectively, these findings demonstrate that pretreatment significantly alters the aroma profile of CLCKO, resulting in clear differentiation in volatile composition between the untreated control and all pretreated groups. Different pretreatment methods induce the formation of differentiated aroma correlation patterns, providing data support for analyzing the regulatory mechanism of pretreatment on CLCKO flavor quality.

### 3.12 Sensory Evaluation

**FIGURE 4:**
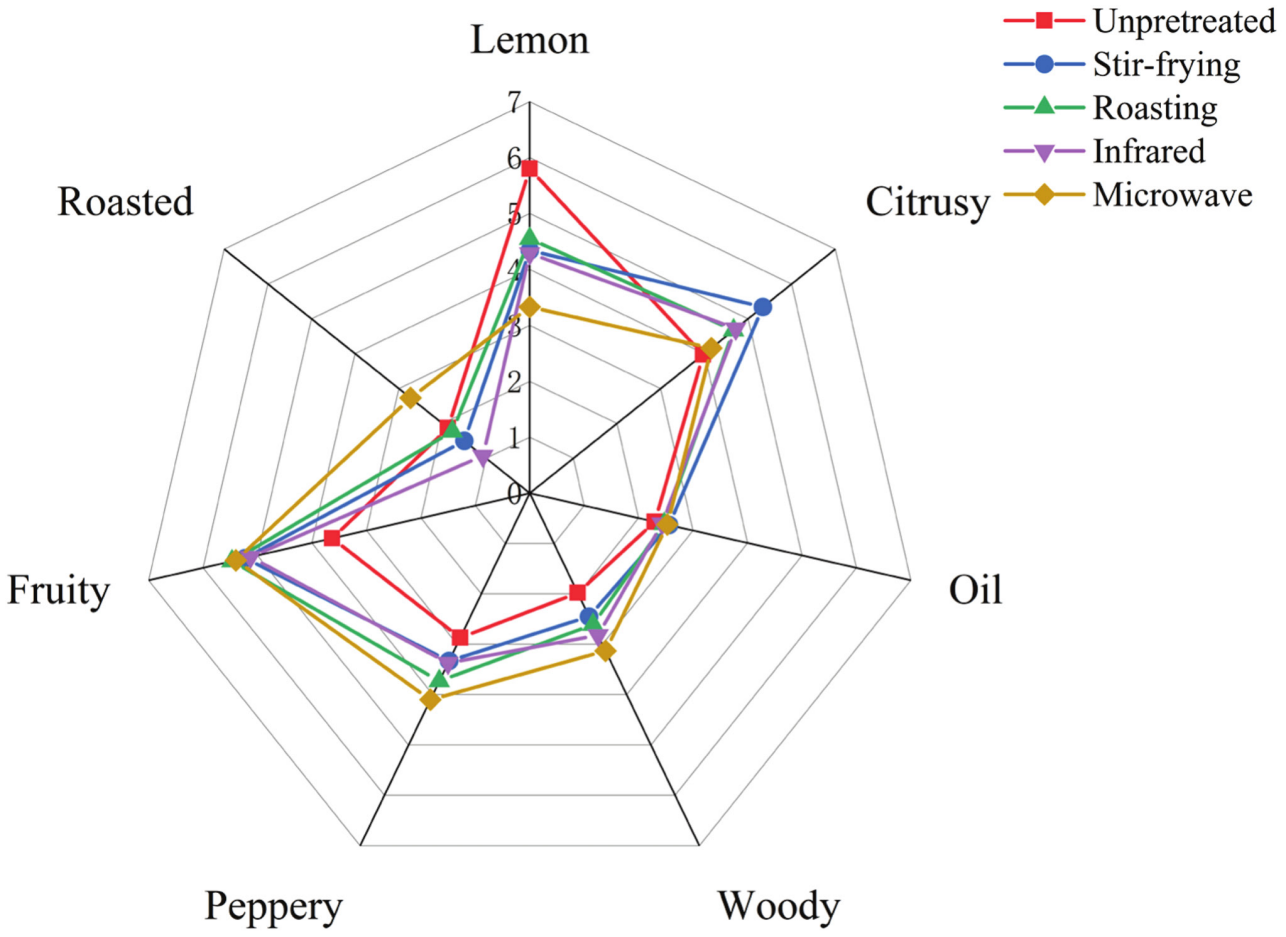
Sensory score chart of CLCKO with different pretreatment.

Due to dynamic variations in the pretreatment process, the sensory characteristics of CLCKO differed across treatment conditions. The sensory evaluations of CLCKO subjected to various pretreatments are shown in Figure 4 The control group exhibited a predominant lemon aroma (5.8). Pretreatments involving stir‐frying yielded CLCKO with dominant citrus (5.34), fruity (5.23), and lemon aroma (4.34). Roasting and infrared‐pretreated groups exhibited fruity aroma (5.13–5.47), citrus aroma (4.67–4.72), lemon aroma (4.29–4.55), and peppery aroma (3.37–3.73). Microwave‐pretreated exhibited sensory attributes dominated by floral‐fruity (5.4), citrusy (4.16), and peppery (4.1) notes, accompanied by subtle lemon (3.33), herbaceous (3.13), and burnt (2.73) aromas. Results indicate that pretreatment increased overall sensory scores, with aroma characteristics exhibiting enhanced floral‐fruity, citrus, and peppery notes.

## 4 Discussion

Pretreatment techniques can enhance oil yield and the content of bioactive compounds while conferring distinctive flavor profiles to oils through thermal reactions such as the Maillard reaction, caramelization, and lipid oxidation (Sun et al. 2025). This study systematically compared the quality implications of various pretreatment methods (stir‐frying, roasting, infrared, and microwave) on CLCKO.

In industrial production, oil yield is a critical determinant of economic viability, whereas physicochemical parameters serve as essential indicators for evaluating the quality and stability of edible oils. The present study demonstrated that all pretreatment methods improved the oil yield of CLCKO. Consistently, Wang et al. (2025) reported a significant increase in the oil extraction rate from safflower seeds following infrared, microwave, and roasting pretreatments, attributing this enhancement to structural disruption of seed tissues and facilitated release of oil‐binding proteins. However, pretreatment methods exerted divergent effects on key oxidative stability indices: acidity value (AV) and peroxide value (POV) varied markedly across treatment groups, with the infrared‐pretreated samples exhibiting the higher AV and POV. Similarly, Alshehri et al. (2024) observed significantly elevated AV and POV in flaxseed oil obtained after infrared pretreatment. Zhang et al. (2025) likewise reported higher AV and POV in walnut oil following infrared treatment, relative to untreated, roasted, and microwave counterparts. Notably, anisidine value (AnV)—a marker of secondary lipid oxidation—was significantly increased in the roasted, infrared, and baked groups in this study. Özdemir et al. (2021) found that roasting hemp seed at 140°C for less than 50 min elevated its AnV. Sundar et al. (2025) reported a comparable AnV increase in perilla seed oil subjected to infrared pretreatment. In contrast, the microwave‐pretreated group exhibited a significant decrease in AnV—a phenomenon potentially attributable to the unique dielectric heating mechanism of microwaves, which may promote the degradation of aldehyde‐type secondary oxidation products and other reactive carbonyl compounds (Zamora et al. 2015). In addition, this study revealed that the fatty acid profile of CLCKO is predominantly composed of linoleic acid and lauric acid. However, its lauric acid content accounts for only approximately 25%, which is markedly lower than the 50% lauric acid content reported previously for LCKO (Cai et al. 2021). This discrepancy may be attributed to differences in oilseed origin, varietal genetic characteristics, and oil extraction process parameters.

Polyphenolic compounds can suppress free radical activity through metal ion chelation, thereby retarding lipid autoxidation (Zieliński et al. 2006). In this study, all pretreatment methods enhanced the total phenolics content of CLCKO, with stir‐frying yielding a significantly higher total phenolics content compared with other pretreatments. In contrast, Ma et al. (2024) reported that microwave pretreatment resulted in a higher total phenolics content in walnut oil than hot‐air or stir‐frying. Such divergent findings may stem from variations in raw material degradation, differences in thermal treatment protocols, and the inherent thermolability of certain phenolic compounds (Huang et al. 2022). Squalene is a characteristic bioactive constituent of edible oils; it exhibits pronounced thermal sensitivity and is highly susceptible to processing conditions. Appropriate pretreatment strategies have been shown to substantially elevate squalene levels, as demonstrated by Gao et al. (2022) and Zhang et al. (2025), who observed increased squalene content in walnut oil following infrared, stir‐frying, microwave, and roasting treatments. Notably, however, this study found a significant decrease in squalene content in CLCKO after pretreatment is potentially attributable to incomplete metabolic conversion (Dąbrowski et al. 2024) or accelerated thermal degradation induced by multiple heating steps (Yang et al. 2025). Furthermore, the DPPH radical scavenging activity of CLCKO ranged from 92.59% to 95.88%, indicating stronger antioxidant capacity than the unpretreated oil following pretreatment. Among the tested methods, infrared and microwave pretreatments conferred the highest antioxidant activity. Similarly, Li et al. (2025) reported that, across various pretreatment methods of roasting, microwave, ultrasound and enzymolysis, the oil clearance rate exceeded 90% at an oil concentration of 80 mg/mL. Microwave pretreatment conferred the strongest antioxidant capacity, which was attributed to its ability to preserve higher levels of tocopherols and total phenolics.

Volatile compounds serve as critical indicators for evaluating aroma quality. Their composition and relative abundance directly govern the characteristic aroma and flavor profiles of oils and fats. Yao et al. (2025) observed that infrared, microwave, and oven heating pretreatments all enhanced the volatile compound content in perilla seed oil, albeit with distinct compositional differences among the treatments: microwave pretreatment preferentially promoted the formation of heterocyclic compounds, whereas infrared and oven heating favored the generation of aldehydes. Likewise, Jiang et al. (2025) demonstrated that thermal pretreatment markedly facilitated the release of flavor‐active volatiles, resulting in elevated concentrations of aldehydes, ketones, and heterocycles in chili oil following stir‐fried, oven‐baked, and microwaved treatment. In the present study, thermal pretreatment similarly enhanced the release of volatile components in CLCKO, with terpenes exhibiting the highest relative abundance and forming the principal structural backbone of the oil's aroma profile. This phenomenon can be ascribed to the physical promotion effect of heat treatment on terpene volatilization. Ni et al. (2021) confirmed that terpenes in *L. cubeba* fruit exhibit poor solubility and limited extractability under low‐temperature conditions; Holopainen and Gershenzon (2010) further elucidated that elevated vapor pressure induced by thermal stress constitutes a key physical driving force underlying the enhanced release of terpenoid compounds from plant matrices. Different volatile compounds impart distinct aroma profiles, thereby contributing to the overall sensory characteristics of the oil. Liu et al. (2025) reported that, relative to untreated peanut oil, both roasting and microwave pretreatment significantly enhanced the formation of key aroma compounds—particularly pyrazines—leading to a pronounced shift in aroma perception from raw bean‐like aroma to roasted, nutty aroma. Similarly, Wang et al. (2023) demonstrated that pyrazine concentrations serve as a reliable indicator of roasting maturity in rapeseed oil during both infrared roasting and traditional roller roasting, correlating strongly with nutty and roasted aromas. In the present study, infrared and roasting promoted the generation of fruity, pungent, sweet, herbal, and rosin‐like aroma notes, whereas microwave and stir‐frying favored the development of lemon, citrus, menthol, and rosin aromas. These differential aroma outcomes are likely attributable to the distinctive volatile compound profile inherent to CLCKO.

## 5 Conclusions

This study systematically investigated the differential effects of four pretreatment technologies—stir‐frying, roasting, infrared, and microwave on the oil yield, physicochemical properties, fatty acid composition, bioactive components, antioxidant capacity, and volatile flavor of CLCKO. Results indicate that, while meeting safety standards, pretreatment significantly increased the oil yield, total phenolic content, and DPPH radical scavenging capacity of CLCKO. It had no significant effect on fatty acid composition. However, all pretreatment methods led to a reduction in squalene content in the oil—albeit to varying extents—underscoring the need to balance enhanced oil yield and phenolic compound extraction with the preservation of squalene. Notably, the infrared‐pretreated group exhibited superior oil yield, total phenolic content, and antioxidant capacity. It also contained richer volatile flavor compounds, with pronounced floral‐fruity, citrus, and peppery notes in sensory evaluation, resulting in a more intense and complex flavor profile.

In summary, infrared pretreatment is a suitable method for enhancing the overall quality of CLCKO, while microwave pretreatment shows potential for flavor diversity. However, as this study aimed to identify the optimal thermal pretreatment technique, future research should focus on further optimizing process parameters, particularly for microwave and infrared pretreatment. Such investigations could provide valuable insights for process optimization and the development of high‐flavor products in industrial production.

## Author Contributions

**Xinsheng Xiao:** writing – original draft, methodology, visualization, data curation, validation. **Yiyang Cao:** software, investigation, validation, methodology, data curation. **Jialing Li:** methodology, software, validation, data curation. **Zhenfeng Jiang:** investigation, formal analysis, conceptualization. **Jinhua Shao:** resources, validation, formal analysis. **Hongyu Jiang:** resources, validation, formal analysis. **Liyan Jiang:** writing – review and editing, funding acquisition, validation, formal analysis, conceptualization.

## Funding

This work was supported by Hunan Provincial Natural Science Foundation of China (2024JJ7192 and 2026JJ80559).

## Conflicts of Interest

The authors declare no conflicts of interest.

## Data Availability

Data will be made available on request.
